# Cardiac sarcoidosis in a patient with ulcerative colitis: A case report and literature review

**DOI:** 10.1097/MD.0000000000036207

**Published:** 2024-01-05

**Authors:** Kentaro Moriichi, Shin Kashima, Yu Kobayashi, Yuya Sugiyama, Yuki Murakami, Takahiro Sasaki, Takehito Kunogi, Keitaro Takahashi, Katsuyoshi Ando, Nobuhiro Ueno, Hiroki Tanabe, Ayumi Date, Sayaka Yuzawa, Mikihiro Fujiya

**Affiliations:** a Division of Gastroenterology, Department of Internal Medicine, Asahikawa Medical University, Asahikawa, Japan; b Division of Cardiology, Nephrology, Pulmonology and Neurology, Department of Internal Medicine, Asahikawa Medical University, Asahikawa, Japan; c Department of Diagnostic Pathology, Asahikawa Medical University Hospital, Asahikawa, Japan.

**Keywords:** cardiac sarcoidosis, interleukin 23, sarcoidosis, Th17, ulcerative colitis

## Abstract

**Rationale:**

Both ulcerative colitis (UC) and sarcoidosis are chronic inflammatory diseases with unknown etiologies and are rare. However, the odds ratio in UC patients has been reported to range from 1.7 to 2.1, suggesting a potential etiology between sarcoidosis and UC. Furthermore, the underlying etiologies of UC and sarcoidosis remain unidentified. Sharing the experience of a UC patient with cardiac sarcoidosis could provide valuable insights to prevent sudden death in UC patients.

**Patient concerns:**

A 71-year-old Japanese woman was diagnosed with UC at 58-year-old and maintained remission on mesalazine treatment. She complained of just palpitation; therefore, she consulted a cardiologist.

**Diagnoses:**

The patient received a diagnosis of cardiac sarcoidosis with complicating ulcerative colitis based on the results of N-terminal prohormone of the brain natriuretic peptide (NT-proBNP), imaging examinations, and histology.

**Intervention:**

The patient was treated with prednisolone and methotrexate. The prednisolone was then tapered, and the methotrexate dose was adjusted based on her symptoms, imaging results, and laboratory findings.

**Outcome:**

She no longer had any symptoms, and the abnormal FDG uptake had disappeared after 2 years.

**Lesson:**

In UC patients, periodic or additional (in case of symptomatic) electrocardiography and NT-proBNP are recommended for the early detection of cardiac sarcoidosis, a life-threatening complication.

## 1. Introduction

Ulcerative colitis (UC) is a chronic bowel inflammatory disease with increasing prevalence worldwide.^[1]^ Despite its unknown etiology, many factors, including psychological distress, autonomic dysfunction, dysbiosis of the gut microbiota, and immunological modulations, are thought to be associated with disease activity.^[2]^ Its extraintestinal complications, such as cutaneous, articular, and hematological abnormalities, have long been recognized.

Sarcoidosis is a rare disease characterized by immunologic anomalies with unknown etiology. Moreover, sarcoidosis and UC are not commonly related. Although 28 patients have been diagnosed with UC associated with sarcoidosis, no cases of UC associated with cardiac sarcoidosis have been reported. In patients with sarcoidosis, the prevalence of cardiac involvement was 20% to 27% and 58% in the United States and Japan, respectively.^[3]^ Cardiac involvement is a main cause of death in patients with sarcoidosis,^[4]^ with sudden death as the leading cause of death in Japan.^[5]^ Thus, to prevent sudden death, cardiac involvement should be identified in patients with sarcoidosis. We present in this report the first case of UC that was associated with cardiac sarcoidosis, which occurred 10 years after the onset of UC.

## 2. Case presentation

A 71-year-old Japanese woman with UC presented to our hospital and complained of palpitation without any other respiratory symptoms, which continued for 7 days; thus, she consulted a cardiologist. The patient was diagnosed with UC at the age of 58 years and maintained remission on mesalazine treatment. Her elder sister underwent surgery for pacemaker insertion due to bradycardia.

Her blood pressure and heart rate were 138/75 mm Hg and 64 bpm with sinus rhythm, respectively. No abdominal symptoms were detected. Laboratory test results on admission are as follows: leukocyte count, 5910/μL; hemoglobin level, 12.3 g/dL; platelet count, 19.9 × 10^4^/μL; erythrocyte sedimentation rate, 8.0 mm/h; C-reactive protein level, <0.1 mg/dL; N-terminal prohormone of the brain natriuretic peptide (NT-proBNP) level, 357.00 (normal range: 0.00–124.99) pg/mL; brain natriuretic peptide level, 88.4 (normal range: ≤18.4) pg/mL; angiotensin-converting enzyme (ACE) level, 10.9 (normal range: 7.7–29.4) IU/L; lysozyme level, 5.5 μg/mL; and soluble interleukin-2 receptor level, 424 (normal range: 122–496) U/mL.

**Figure 1.**
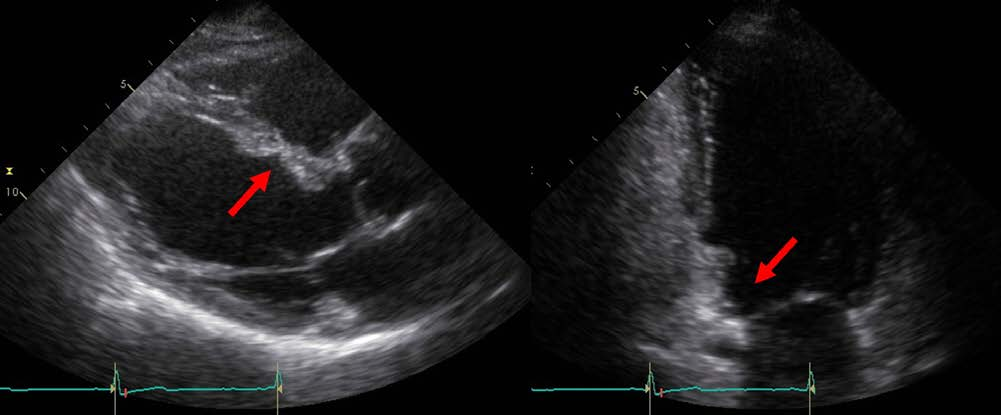
Ultrasound echocardiographic images. Ultrasound echocardiography revealed thinning of the midanteroseptal (left) and basal inferior (right) walls (arrow).

**Figure 2.**
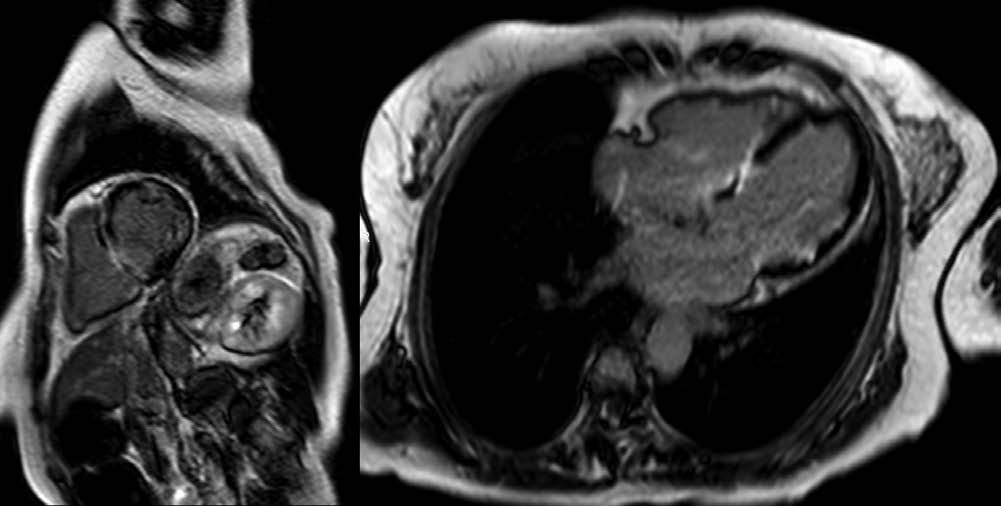
Cardiovascular magnetic resonance images. Cardiovascular magnetic resonance imaging revealed late gadolinium enhancement from the basal to midwalls of the left ventricle (left), as well as focal gadolinium enhancement on the midseptal and lateral walls (right).

**Figure 3.**
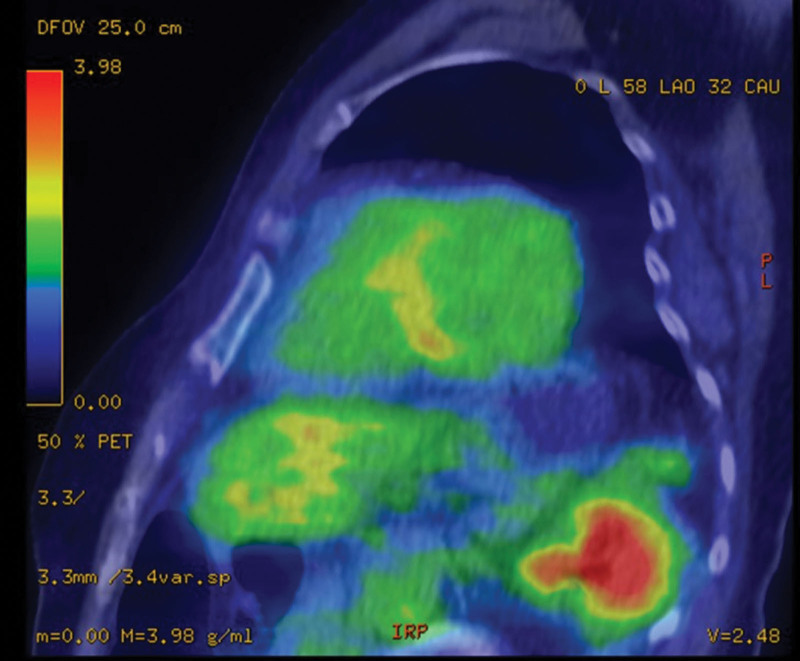
Positron emission tomography and computed tomography (PET/CT) scan image. PET/CT scan showed fluorodeoxyglucose uptake from the basal to mid inferoseptal and inferior walls.

**Figure 4.**
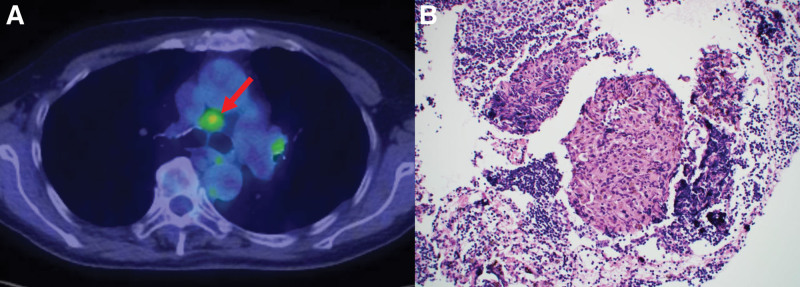
PET/CT scan image of the subcarinal lymph node and pathological findings of the lymph node. (A) PET/CT scan demonstrated the accumulation of fluorodeoxyglucose (FDG, arrow) in the subcarinal lymph node (#7). (B) A lymph node biopsy specimen taken from the subcarinal lymph node showed noncaseating epithelioid granulomas. PET/CT = positron emission tomography and computed tomography.

Screening electrocardiography (ECG) and Holter ECG were performed because of investigation of palpitation. Although ECG didn’t detect any arrythmia, Holter ECG revealed frequent premature atrial contraction and premature ventricular contraction. On the same day to exclude organic diseases, echocardiography was performed and revealed hypokinesis or akinesis in the left ventricular inferoseptal and basal inferior walls and thinning of the mid anteroseptal and basal inferior walls (Fig. 1). These findings suggested the possibility of cardiomyopathy including cardiac sarcoidosis. For further investigations, additionally magnetic resonance imaging of the cardiovascular system examination and positron emission tomography and computed tomography (PET/CT) were also performed. magnetic resonance imaging of the cardiovascular system revealed late gadolinium enhancement on the left basal to the midventricular wall (Fig. 2). PET/CT scan revealed a slightly decreased FDG uptake in the left ventricular myocardium (Fig. 3). Based on the results of these examinations, cardiac sarcoidosis was highly suspected. To make final diagnosis, cardiac biopsy was performed. Although cardiac biopsy samples revealed no specific abnormalities, PET/CT scan demonstrated FDG accumulation in the subcarinal lymph node (Fig. 4A), and lymph node biopsy samples revealed noncaseating granulomas (Fig. 4B).

**Figure 5.**
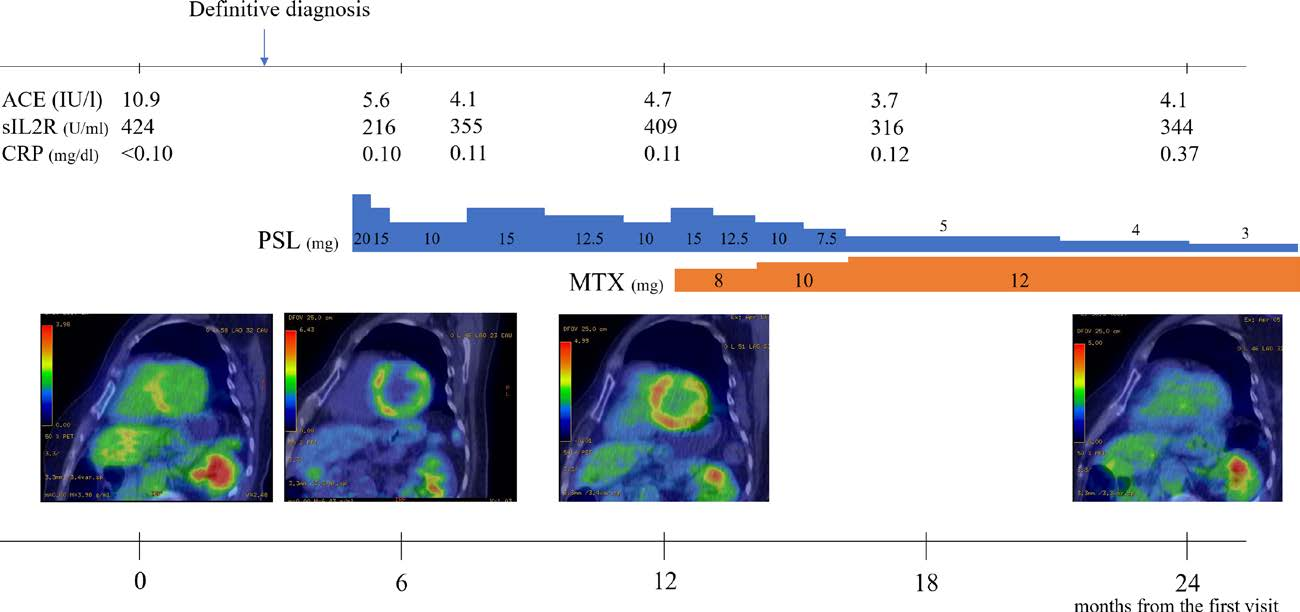
Clinical course. A 20 mg of prednisolone (PSL) was administered daily. However, after its administration, PET/CT scan revealed increased FDG uptake in the cardiac muscles at 6 and 12 months after the first visit. Therefore, 8 mg/day of methotrexate (MTX) was added to the regimen, and the PSL dose was increased to 15 mg/day. The PSL was then tapered, and the MTX dose was adjusted. Her symptoms and the abnormal FDG uptake detected via PET/CT scan 2 years after her first visit had disappeared. FDG = fluorodeoxyglucose, PET/CT = positron emission tomography and computed tomography.

These imaging findings were consistent with the cardiac sarcoidosis criteria (Table 1),^[6]^ and consequently, the patient was diagnosed with cardiac sarcoidosis. Generally, to reduce inflammation, immunosuppressive and/or immunomodulatory drugs are used for controlling the disease.^[7]^ Corticosteroid treatment for cardiac sarcoidosis widely accepted.^[8]^ A 20 mg of prednisolone (PSL) was initiated daily. However, PET/CT scan during PSL tapering 1 year after the first visit revealed higher FDG uptake in the cardiac muscles than the FDG uptake before PSL treatment (Fig. 5). At that time, we thought it was difficult to taper PSL and continue PSL monotherapy. Methotrexate (MTX) is known as the most common steroid-sparing agent.^[8]^ Thus, an 8 mg/wk of MTX was added to the regimen, and the PSL dose was increased to 15 mg/d. The PSL was then tapered, and the MTX dose was adjusted based on the patient’s symptoms, imaging results, and laboratory findings.

**Table 1:** Criteria for the Diagnosis of Cardiac Sarcoidosis (CS).

There are 2 pathways to a diagnosis of CS: 1. Histological Diagnosis from Myocardial Tissue CS is diagnosed in the presence of non-caseating granuloma on histological examination of myocardial tissue with no alternative cause identified (including negative organismal stains if applicable). 2. Clinical Diagnosis from Invasive and Non-Invasive Studies: It is probable* that there is CS if: a) There is a histological diagnosis of extra-cardiac sarcoidosis and b) One or more of following is present •Steroid +/- immunosuppressant responsive cardiomyopathy or heart block •Unexplained reduced LVEF (<40%) •Unexplained sustained (spontaneous or induced) VT •Mobitz type II 2nd degree heart block or 3rd degree heart block •Patchy uptake on dedicated cardiac PET (in a pattern consistent with CS) •Late Gadolinium Enhancement on CMR (in a pattern consistent with CS) •Positive gallium uptake (in a pattern consistent with CS) and c) Other causes for the cardiac manifestation(s) have been reasonably excluded

PET = positron emission tomography.

* In general, ‘probable involvement’ is considered adequate to establish a clinical diagnosis of CS.

No symptoms were observed after administering MTX, and the abnormal FDG uptake detected via PET/CT scan 2 years after her first visit had disappeared (Fig. 5). Moreover, no abdominal symptoms were observed during the therapy, and she maintained endoscopic remission.

## 3. Discussion

To the best of our knowledge, this is the first report of cardiac sarcoidosis in a patient with UC. Diagnosing cardiac sarcoidosis is critical considering that it is the main cause of death in patients with sarcoidosis and associated with poor prognosis.^[3,4]^ The characteristics of cardiac sarcoidosis must be assessed to prevent the occurrence of sudden death in patients with UC. Nonetheless, diagnosing cardiac sarcoidosis is extremely challenging because it has not yet been reported in patients with UC. Therefore, to detect the early stages of cardiac sarcoidosis in UC patients, periodic or additional (in case of symptomatic) examinations such as electrocardiography and NT-proBNP are recommended.

It seems very hard to prove association with cardiac sarcoidosis in UC patients. However, in patients with UC, the odds ratio for sarcoidosis was reported to be 1.7 (95% confidence interval [CI]: 1.2–2.2) to 2.1 (95% CI: 1.2–2.2)^[9,10]^ and the high incidence of cardiac involvement in sarcoidosis patients, implying a potential etiological association between sarcoidosis and UC.

Sarcoidosis is characterized by a persistent granulomatous reaction to an unknown antigen, which is believed to be triggered by Th1 cells.^[11]^ Recently, Th17 cells, which are developed in response to IL-23 and IL-1β, are implicated in the pathogenesis of sarcoidosis.^[12]^ Th17 cells have been found surrounding and within the lung granuloma during the early and progressive phases of sarcoidosis with the development of fibrotic changes, suggesting that Th17 may accelerate the course of sarcoidosis.^[12]^ Furthermore, Th17 has been implicated in the pathogenesis of UC. In patients with UC, IL-17 mRNA and IL-23 receptor mRNA expression and RORC mRNA expression were all significantly upregulated in the intestinal lamina propria (LP) CD4 + cells. Recently, IL-23 pathway plays a critical role in various chronic inflammatory diseases, such as inflammatory bowel disease, psoriasis, multiple sclerosis, and arthritis.^[13]^ The role of IL-23 in Th17 cell has not yet been fully elucidated and the mechanism of Th17 differentiation regulated by IL-23 has been still controversial, however, IL-23 is thought to affect Th17 cell which can produce IL-17A, IL-17F, IL-21, and IL22 expansion, stabilization, and/or conditioning for a fully inflammatory cell phenotype.^[13]^ IL-23 induced a significant IL-17 upregulation in LP CD4 + cells isolated from patients with UC.^[14]^ Furthermore, immunohistochemical staining of the colonic mucosa of patients with UC revealed an increased expression of IL-17 positive cells.^[15,16]^ These studies suggest that IL-17 plays a crucial role in the pathogenesis of both sarcoidosis and UC. Thus, the interleukin (IL)-23/Th17 pathway has been identified to play a critical role in several chronic inflammatory diseases including inflammatory bowel disease.^[17]^

Only 28 cases of UC, including this case, have been reported with concomitant sarcoidosis affecting various organs, including the lungs and other organs but not the heart (Table 2).^[18–37]^ Three of them were reported to be drug-induced sarcoidosis resulting from the use of tumor necrosis factor-alpha (TNF-α) antagonists.^[22–24]^ Moreover, various drugs, including TNF-α antagonists, interferon or pegylated interferon therapies, BRAF or MEK inhibitors, and immune checkpoint inhibitors, are associated with the development of sarcoidosis.^[38]^ However, no TNF-α antagonists or possible sarcoidosis-inducing medications have been administered in the present case. Sarcoidosis developed after the UC diagnosis in 16 cases, including the present case, who had not received any medications known to cause sarcoidosis.^[20,25–37]^ In these cases, the average age at onset of sarcoidosis was 39.5 ± 14.3 years, and the male-to-female ratio was 10:6, with the mean time interval between the UC and sarcoidosis onset being 156.8 ± 105.7 months. Ten cases developed sarcoidosis > 10 years after the UC diagnosis, and 13 cases had chest lesions, including lung, hilar, and/or mediastinal lymphadenopathy. On admission for relapsing UC without respiratory symptoms, half of the cases with chest lesions were accidentally diagnosed with sarcoidosis. However, only 5 cases had respiratory symptoms, such as dry cough, chest pain due to pleuritis, and dyspnea, indicating difficulties in diagnosing sarcoidosis. Interestingly, 8 cases (50%) underwent proctocolectomy (7 cases) or subtotal colectomy (1 case) due to deteriorating UC. These findings imply that sarcoidosis may affect the duration and/or severity of UC. The prognosis of sarcoidosis was reported in 9 cases of UC who all had improved or completely resolved lesions. Five of these 9 cases required steroid treatment to manage sarcoidosis. These findings suggest that a favorable prognosis can be expected when sarcoidosis is properly diagnosed and treated.

**Table 2:** Summary of 28 cases with sarcoidosis in UC patients.

Case	Type	Type of UC	Sarcoidosis onset age	Gender	Interval between the onset of UC and sarcoidosis (months)	Sarcoidosis related symptoms	Thoracic lesions				Extrathoracic lesions	Prognosis of Sarcoidosis		Ref
							Lymphadenopathy		Lung field infiltration	Others		Improvement	Duration till improvement (months)	
							Hilum	Media- stinum						
1	SAR first	Total	52	F	156	Dyspnea	Yes					NA		14
2	SAR first	Proctitis	64	M	144	Irritating area on his scalp, skin eruption					Scalp, Iritis	Yes	NA	16
3	SAR first	Proctitis	38	F	72	Fever, myalgia and chest pain.			Yes	Yes		Yes	6	16
4	SAR first	Total	33	M	180	Retinal vasculitis						Yes	NA	16
5	SAR first	Proctitis	37	F	108	Erythema nodosum	Yes				Muscle	Yes	36	16
6	Drug induced	Total	68	F	23	Previous scars and several painful dermal nodules						Yes	NA	18
7	Drug induced	NA	42	M	252	Dyspnea, dry cough, low-grade fever			Yes		Kidney	Yes	NA	19
8	Drug induced	Total	30	M	60	Dyspnea, nocturnal sweating, nonproductive cough		Yes	Yes		Retroperitoneal adenopathies	Yes	NA	20
9	UC first	Total	30	M	12	None	Yes	Yes			Lymphnode in the porta hepatis	NA		21
10	UC first	Total	26	M	98	None		Yes			Ileocecal lymph nodes	NA		22
11	UC first	Proctitis →total	44	F	240	Dry cough, SB, fever up, pleuritic chest pain, BWL			Yes			Yes	24	16
12	UC first	total	20	M	192	Iritis	Yes	Yes			Iritis	Yes	NA	16
13	UC first	Total	47	M	156	Fever, Myalgia, BW loss, Hepatosplenomegaly					Liver	Yes	NA	16
14	UC first	Total	30	M	156		Yes	Yes			Lymphnode in the porta hepatis	NA		23
15	UC first	NA	42	M	216							NA		24
16	UC first	Total	38	M	216	High fever, BWL	Yes					NA		25
17	UC first	Cecum + proctsigma	41	M	48			Yes				Yes	34	26
18	UC first	NA	58	F	432	Skin leions					Fingers, hands, temples	NA		27
19	UC first	NA	22	M	72	Cervical lymphadenopathy	Yes				Cervical lymphadenopathy	NA		28
20	UC first	NA	38	F	72	Chronic productive cough, low grade fever, SB.	Yes					Yes	NA	29
21	UC first	Left	52	M	84	Low grade fever, BWL, chills, palpable mass, cough, breathness	Yes	Yes				Yes	1	30
22	UC first	Total	33	F	120	Subcutaneous masses	Yes				Forearm	NA		31
23	UC first	NA	50	F	108	Breathlessness	Yes	Yes	Yes			Yes	18	32
24	UC first	NA	62	F	288	Dry cough	Yes	Yes			Hip, forearms, and legs	Yes	24	33
25	UC first	Total	71	F	156	Palpitation		Yes				Yes	24	our case
26	Same time	Total	64	M	0	None	Yes	Yes	Yes			Yes	16	15
27	Same time	Proctsigma	31	M	0	Dry cough, SB.	Yes					Yes	9	16
28	Same time	proctitis	33	M	0	Dry cough	Yes					Yes*	24	17

BWL = body weight loss, SB = shortness of breath, UC = ulcerative colitis.

* Remaining obstructive ventilatory defect.

Several plausible explanations have been considered for the lack of previous reports of cardiac sarcoidosis in patients with UC. First, cardiac sarcoidosis is difficult to identify in asymptomatic patients. Second, clinical evidence of cardiac involvement has been reported in only 5% of patients with sarcoidosis.^[4]^ Third, although serum ACE levels have been associated with the occurrence of sarcoidosis, the prevalence of elevated ACE levels in patients with sarcoidosis has been reported to be only 21.8%, indicating the absence of sensitive markers available to predict sarcoidosis.^[39]^ Fourth, the histologic diagnostic rate of cardiac sarcoidosis was inadequate. For instance, Uemura et al^[40]^ performed right ventricular biopsies from 26 patients with a strong suspicion of cardiac sarcoidosis, and only 5 cases (19.2%) revealed a characteristic finding, i.e., noncaseating granulomas. Fifth, patients with UC have not been thoroughly screened for sarcoidosis since concomitant UC and sarcoidosis extremely rarely developed. These factors appear to hinder the diagnosis of cardiac sarcoidosis. The prognosis of cardiac sarcoidosis was thought to be related with heart failure.^[41]^ To improve the prognosis of cardiac sarcoidosis, early detection is warranted. Although it was reported high prevalence of atrio-ventricular block as the initial symptom^[3]^ and ventricular tachycardia^[42]^ of cardiac sarcoidosis, as the majority of cases were asymptomatic, periodic examinations such as electrocardiography, NT-proBNP appear to be effective in the early detection of cardiac sarcoidosis. Because mediastinal lymphadenopathy was detected in one-third of patients with UC and sarcoidosis, chest CT scans should be considered when necessary if these examinations show no abnormal findings. This will help detect and improve the outcomes of cardiac sarcoidosis in patients with UC.

There are several limitations of this report. First, since this is the first case report of cardiac sarcoidosis in UC patients, while a summary of UC patients with sarcoidosis (excluding cardiac sarcoidosis) would be helpful to understand their characteristics, it is possible that the characteristics are not fully reflected in this report. Second, the etiology of this case remains unclear. Although both UC and sarcoidosis are thought to be associated with the same factors such as Th17 and IL-23, it cannot be proven how these elements are related so far.

Taken together, average period of developing sarcoidosis is more than 10 years after the UC diagnosis. It predominantly affects males, and in most cases, mediastinal lymphadenopathy is observed. To detect cardiac sarcoidosis in early stage, electrocardiography and NT-proBNP are recommended. Although the etiology remains unknown, common underlying causes is thought to be existed because similar factors are associated with both UC and sarcoidosis.

## 4. Conclusion

This is the first report of cardiac sarcoidosis occurring in a patient with UC. The immunologic abnormality associated with IL23–Th17 cells was thought to be involved in the etiology of both disorders. Although cardiac sarcoidosis very rarely occurs in patients with UC, it is a life-threatening condition; therefore, periodic cardiac examinations, such as electrocardiography and NT-proBNP, are still recommended to identify the occurrence of cardiac sarcoidosis even with the absence of chest symptoms.

## Acknowledgments

All authors have read the journal’s policy on conflicts of interest and authorship agreement.

## Author contributions

**Conceptualization:** Kentaro Moriichi, Shin Kashima.

**Data curation:** Kentaro Moriichi, Shin Kashima, Yu Kobayashi, Yuya Sugiyama, Yuki Murakami, Takahiro Sasaki, Takehito Kunogi, Keitaro Takahashi, Hiroki Tanabe, Ayumi Date, Sayaka Yuzawa.

**Methodology:** Shin Kashima, Mikihiro Fujiya.

**Supervision:** Kentaro Moriichi, Mikihiro Fujiya.

**Validation:** Kentaro Moriichi, Shin Kashima, Katsuyoshi Ando, Nobuhiro Ueno, Mikihiro Fujiya.

**Writing – original draft:** Kentaro Moriichi, Shin Kashima, Mikihiro Fujiya.

**Writing – review & editing:** Kentaro Moriichi, Shin Kashima, Mikihiro Fujiya.
